# Toxoplasmic retinochoroiditis: The influence of age, number of retinochoroidal lesions and genetic polymorphism for IFN-γ +874 T/A as risk factors for recurrence in a survival analysis

**DOI:** 10.1371/journal.pone.0211627

**Published:** 2019-02-12

**Authors:** Ana Luisa Quintella do Couto Aleixo, Raquel Vasconcelos C. de Oliveira, Maíra Cavalcanti Albuquerque, Ana Luiza Biancardi, André Luiz Land Curi, Eliezer Israel Benchimol, Maria Regina Reis Amendoeira

**Affiliations:** 1 Infectious Ophthalmology Laboratory, Evandro Chagas National Institute of Infectious Diseases—FIOCRUZ, Rio de Janeiro, Brazil; 2 Evandro Chagas National Institute of Infectious Diseases—FIOCRUZ, Rio de Janeiro, Brazil; 3 Laboratory, Oswaldo Cruz Institute –FIOCRUZ, Rio de Janeiro, Brazil; 4 Universidade Federal do Rio de Janeiro -UFRJ, Rio de Janeiro, Brazil; Oregon Health and Science University, UNITED STATES

## Abstract

**Purpose:**

To analyze risk factors for recurrent toxoplasmic retinochoroiditis.

**Design:**

Single center prospective case series.

**Population and Methods:**

A total of 230 patients with toxoplasmic retinochoroiditis were prospectively followed to assess recurrences. All patients were treated with a specific drug regime for toxoplasmosis in each episode of active retinochoroiditis. Individuals with chronic diseases and pregnant women were excluded. Survival analysis by extended Cox regression model (Prentice-Williams-Peterson counting process model) was performed to evaluate the time between recurrences according to some potential risk factors: age, number of retinochoroidal lesions at initial evaluation, sex and interferon gamma +874 T/A gene polymorphism. Hazard Ratios (HR) and 95% confidence intervals (CI) were provided to interpret the risk effects.

**Results:**

One hundred sixty-two recurrence episodes were observed in 104 (45.2%) patients during follow-up that lasted from 269 to 1976 days. Mean age at presentation was 32.8 years (Standard deviation = 11.38). The risk of recurrence during follow up was influenced by age (HR = 1.02, 95% CI = 1.01–1.04) and number of retinochoroidal lesions at the beginning of the study (HR = 1.60, 95% CI = 1.07–2.40). Heterozygosis for IFN-γ gene polymorphism at position +874 T/A was also associated with recurrence (HR = 1.49, 95% CI = 1.04–2.14).

**Conclusion:**

The risk of ocular toxoplasmosis recurrence after an active episode increased with age and was significantly higher in individuals with primary lesions, which suggests that individuals with this characteristic and the elderly could benefit from recurrence prophylactic strategies with antimicrobials. Results suggest an association between IFN-γ gene polymorphism at position +874T/A and recurrence.

## Introduction

Toxoplasmosis is widely distributed and has high prevalence around the world. In Brazil, the prevalence of ocular toxoplasmosis varies according to the area under study and can reach 17.7% of the population [1,2]. Individuals affected by toxoplasmic retinochoroiditis (TRC) are at risk of recurrent episodes throughout their lives, which may cause visual impairment. [3,4] Recurrence studies are extremely difficult to perform, mainly because they demand long follow-ups and also because recurrences may be influenced by an intricate network of possible and still unknown protection and risk factors. There are indications of a clustering pattern of recurrences and the course of the disease may be related to individual’s age, transmission form, whether congenital or acquired, virulence of *T*. *gondii* strain and host susceptibility. [5,6,7]

Some researches indicate host immunogenetic aspects as potential protection [8] or risk factors for TRC. [9] Immunologic mechanisms against infection by *T*. *gondii* and particularly its latency are not entirely understood, yet it has been suggested that an intense TH 1 response, with interferon gamma secretion is critical for host protection. [10,11] Genetic polymorphism of genes that synthesize interferon- gamma (IFN-γ) might be related to phenotypes with higher or lower response in IFN-γ production, which is possibly associated with the resistance or susceptibility to some infectious diseases including TRC. [12,13]

Despite the negative impact of recurrence on the vision prognosis of TRC patients, there are few papers published on this characteristic of the disease related to potential triggering or individual susceptibility factors. From the clinical point of view, the paper published by Felix et al. in 2014 promoted the use of prophylactic antimicrobials to prevent recurrence. They demonstrated in a double-masked randomized clinical trial the benefit of this strategy in TRC treatment, confirming the evidence found by Silveira et al. in 2002. [14,15] Studying TRC recurrence is essential to guide the use of drug prophylaxis, helping to determine which group of individuals could really benefit from this strategy.

The purpose of this study was to analyze TRC recurrence after an episode of active disease, identifying characteristics of the individuals at higher risk and a possible association with genetic polymorphism of the gene that synthesizes IFN-γ at position +874 T/A.

## Patients and methods

Patients with toxoplasmic retinochoroiditis were enrolled in the outpatient unit of Infectious Ophthalmology Laboratory of Evandro Chagas National Institute of Infectious Diseases (INI)- Fiocruz, from January 2010 to January 2014 and were followed until July 2015. The study was approved by the Evandro Chagas National Institute of Infectious Diseases (INI)Ethics Committee (CAAE 0075.0.009.00011) and all patients signed an informed consent form. Subjects between 14 and 18 years old who agreed to participate also signed an informed consent in conjunction with a parent or surrogate. Detailed description of the formation of this cohort and its characteristics have been previously published [16].

The study included patients with positive serology for *Toxoplasma gondii* and a clinical diagnosis of active toxoplasmic retinochoroiditis according to the criteria described by Holland. [17] Recurrent cases were defined as an active retinochoroiditis associated with retinal scar in either eye. [18] Episodes of inflammation of the anterior segment in eyes with retinochoroiditis scars were not considered recurrence. [17] Creamy-white focal retinochoroidal lesions in the absence of other retinochoroidal scars were considered as primary lesions. Primary retinochoroidal lesion cases with no recurrence were considered to be highly probable of ocular toxoplasmosis and thus included in the follow-up.

Exclusion criteria comprised conditions such as: pregnant women, chronic renal failure, AIDS, syphilis, report of intravenous drug use and also patients under chemotherapy or immunosuppressive drugs, peri or intra-ocular corticosteroid treatment, transplanted or those with manifest immunosuppression, diagnosed autoimmune disease and–multifocal active retinochoroidal lesions. Patients with single, unilateral active retinochoroidal lesions were submitted to serologic tests for syphilis and HIV at INI Immunology and Immunogenetic Laboratory. Those with positive results or those who did not respond to toxoplasmosis treatment during the first 45 days were excluded.

All patients with active retinochoroidal lesions were treated with a standardized drug regimen over 30 to 45 days (1g q6h sulfadiazine, 25–50 mg/day pyrimethamine, 15 mg 3x/week folinic acid and 0.5 to 1 mg/kg/day prednisone as initial dose followed by progressive tapering) or clindamycin (300 mg q6h) replacing sulfadiazine in case of allergy and alternatively with 800mg-160mg q12h sulfametoxazole/ trimethoprim with tapering prednisone.

The method described by Pravica et al. (2000) was used to assess polymorphism in IFN-γ, at the +874 T/A position, with the target genomic DNA fragment amplified by PCR, using oligonucleotides specific for IFN-γ +874. [19] Genomic DNA was extracted using a commercial extraction kit (Qiagen, Hilden, Germany), following the manufacturer’s protocol.

### Statistical analysis

Exploratory data analysis was carried out through summary measures such as mean, standard deviation (SD), median, minimum and maximum of quantitative variables and frequency of qualitative variables. Confidence interval (95% CI) was used to describe uncertainty of summary measures, such as, proportions.

Survival analysis was performed by the Cox regression model considering the time to each recurrence (event) during follow-up. Counting process was considered to model the time. Censoring was defined by absence of recurrence until the date of the last visit. This analysis was performed using an extended Cox regression for multiple events (conditional model of Prentice, Williams and Peterson, 1981 –PWP) [20] to estimate the effects associated with time until recurrences. PWP is a model that considers multiple events and is able to incorporate the ordered events (recurrences) in estimation of concomitant risk. For this purpose, the PWP model stratified the baseline risk based on the number of the recurrent event (first recurrence, second, third, and so on) and added a cluster effect by patient.

The following characteristics were considered as potential recurrence risk factors: sex, age at study admission, number of retinochoroidal lesions and genetic polymorphism of IFN-γ +874 T/A gene. Initially, we estimated the effect of each variable over time until recurrences by single covariate (simple) model. After that, a multiple covariate model was performed to adjust the effects of variables.

The incidence rate was represented by the Hazard Ratios (HR) obtained by the simple model (crude HR) and the multiple model (adjusted HR), with their respective 95% confidence intervals (CI). The values of crude HR were compared to those of adjusted HR to check for possible modifying effects or confounding. Model assumptions such as proportionality and linearity were assessed by Schoenfeld residuals analysis and splines.

Statistical tests were performed by R statistical software version 3.1.2 (R: A Language for Statistical Environment Computing, R Core Team, Vienna, Austria, 2014).

## Results

We analyzed 162 recurrence episodes in 104 out of 230 patients monitored over periods that varied from 269 to 1976 days, mean 1060 days. The main characteristics of the studied population are summarized in Table 1.

**Table 1:** Characteristics of 230 toxoplasmic retinochoroiditis patients, Rio de Janeiro, July 2015.

Recurrences during follow-up	Yes (N = 104)			No (N = 126)		
	n	%	95% CI	N	%	95% CI
**Sex**						
Female	51	49.0	39.10–59.03	61	48.4	39.41–57.49
Male	53	50.9	40.97–60.90	65	51.6	42.51–60.59
**Toxoplasmosis serology**						
Only IgG	93	89.4	81.87–94.60	116	92.1	85.89–96.12
IgG and IgM	11	10.6	5.40–18.13	10	7.9	3.88–14.11
**Number of retinochoroidal lesions**						
Primary lesion	33	31.7	22.94–41.59	19	15.1	9.32–22.54
Recurrent lesion (2 or more)	71	68.3	58.41–77.05	107	84.9	77.46–90.68
**IFN-γ +874 gene polymorphism**						
AA	35	33.6	24.69–43.59	64	50.8	41.73–59.81
AT	50	48.1	38.18–58.09	41	32.5	24.47–41.45
TT	19	18.3	11.38–27.05	21	16.7	10.62–24.34

There was a homogenous sex distribution with mean age of 32.8 years (SD = 11.38, range 14 to 77 years). The IgG and IgM toxoplasmosis serology had a similar distribution between individuals that had recurrences and those that did not.

In the PWP multiple modeling, considering all 230 patients and their recurrences (162 events in 104 patients) and adjusted by sex, three variables appeared as significant factors: age, heterozygosis for IFN-γ at position +874 T/A and number of lesions. The risk of recurrence increased 2% for each increase of 1 year of age (HR = 1.02) if controlled by the other variables. Patients with AT heterozygosis for IFN-γ +874 T/A gene had 49% greater risk of recurrence than those with AA homozygosis and patients with just one lesion presented 60% greater risk than those with more than 2 lesions (Table 2). There was no evidence of confusion or effect modification factors, since the values of adjusted HR are close to those of crude HR (Table 2). The multi covariate model presented proportionality in all the variables according to Schoenfeld residuals and the inclusion of a non-linear term for age (spline) showed no rejection of linearity.

**Table 2:** Simple and multiple PWP* model for time until each recurrence in 230 patients (162 events in 104 patients) with toxoplasma retinochoroiditis diagnosis, Rio de Janeiro, July 2015.

Variable	Category	Crude-HR (95% CI)	Adjusted-HR (95% CI)
Sex	Male	1.15 (0.84–1,57)	1.19 (0.86–1.64)
	Female	1.00	1.00
Age (years)	-	**1.03** **(1.01–1.04)**	**1.02** **(1.01–1.04)**
IFN-γ +874 T/A gene polymorphism	AT	**1.55** **(1.09–2.21)**	**1.49** **(1.04–2.14)**
	TT	1.24 (0.81–1.94)	1.27 (0.80–2.01)
	AA	1.00	1.00
Number of lesions	1	**1.61** **(1.09–2.37)**	**1.60** **(1.07–2.40)**
	2	1.27 (0.86–1.88)	1.17 (0.79–1.73)
	More than 2	1.00	1.00

Note: HR- Hazard Ratio, CI- Confidence Interval, P<0,05 in bold,

*conditional model of Prentice, Williams and Peterson, 1981—considering all recurrence observations over time

## Discussion

Studying the factors that influence recurrence of ocular toxoplasmosis is challenging due to multiple factors potentially involved. This study evaluated prospectively a large number of TRC patients, treated with a standardized drug regimen, selected with pre-determined diagnostic criteria and examined by the same ophthalmologists at every visit. This method, associated with the survival analyses for host risk factors performed, distinguishes it from the available scientific literature on this disease. In this cohort, recurrence risk after a TRC active episode was significantly higher in patients with primary lesion (defined as an active focal lesion in the absence of other retinochoroidal scars) and increased with patient’s age. This result is in agreement with previous literature data from retrospective studies, which indicates that the elderly and patients with a primary lesion have higher risk of recurrence. [21,22]

Most cases of TRC observed in the follow-up of this population are concentrated in young and middle-aged adults, a fact that had already been reported.

[4,23,24] This creates the false impression that recurrence risk is higher among young people, since the frequency of toxoplasmosis episodes is higher in this group. The age at the time of the first episode or of the toxoplasma infection has been considered as a recurrence risk factor. The moment of toxoplasma infection could not be analyzed in this study due to our methodology of patient selection, so it was unable to find out whether the duration of the infection plays a role in the recurrence risk. We observed that the first symptomatic or observed episode has a greater probability of being a recurrence, because most patients have old healed scars from previous asymptomatic episodes. [16,24]. This aspect deserves further studies, as previous non-treated episodes could influence recurrence risk. A study with several patients in the Netherlands showed that recurrence risk was higher immediately after an episode of active disease, then decreased with increasing disease-free intervals, influenced by patient age at the first TRC episode and patient age at the time of reactivation. In addition, patients older than 40 years of age, at the time of an active episode, were at higher risk of recurrence than younger patients.[7,21,24] Another study, indicated a smaller risk of recurrence in individuals older than 40 years of age [25], which could be explained by a probable protective effect of a greater disease-free interval and the age at the first episode. [7] Recently another publication confirmed the results of the Dutch cohort, also suggesting that TRC risk of recurrence is higher during the first year after an active episode and increases with the patient’s age [22].

The results of this study show a linear relationship between aging and risk of recurrence, which can help to guide the indications for antimicrobial prophylaxis. In 2002 Silveira et al. described the possibility of using antimicrobial prophylaxis to reduce recurrence and recently Felix et al. showed that the use of sulfamethoxazole and trimethoprim prevented TRC recurrence in patients with healed lesion associated with active lesion. [14,15].

Investigating this prevention strategy in patients with primary retinochoroidal lesion is critical, because there is an indication that the risk of recurrence is higher during the first year monitoring these patients. [22] The relationship between age and recurrence should raise questions on which immunological aspect of senescence could be influencing retinal latency of *T*. *gondii* cysts, its persistency and replication that can lead to recurrences. There are indications that the patient’s age at the time of infection and its congenital or acquired origin may influence the risk of recurrence, however, it was not possible to determine the frequency of this characteristic in the present study. The presence of IgM antibodies in only 21 of 230 (9.1%) patients indicates acquired infection and this aspect was not included in the statistical model as it would be insufficient to analyze the small number of individuals with the chosen methodology.

IFN-γ is a cytokine considered crucial in the immune response against *T*. *gondii* because it contributes to the control of tachyzoite proliferation *in vivo*, however its role in inducing and maintaining bradyzoites in cystic form is still not clear. [10,11,26] It is possible that IFN-γ is directly related to suppressing recurrences and maintaining cystic bradyzoites in a latent state. The association of IFN-γ +874T/A gene polymorphism with higher or lower IFN-γ production could represent a greater susceptibility to ocular toxoplasmosis, its recurrences and more severe forms of systemic toxoplasmosis. Neves et al. showed in a control case study that TT genotype could be associated with a greater risk of evolution to more severe forms of acute acquired toxoplasmosis, but the 30-individual group analyzed was considered too small to demonstrate correlation between polymorphism and retinochoroiditis. [27].

Recurrence is a characteristic aspect of toxoplasmic retinochoroiditis (TRC) and is generally required to establish a definite diagnosis of the disease. [23] Because of that, individuals with primary lesion were only included in this study if they showed healing of retinochoroiditis after 30 to 45-day treatment with a toxoplasma specific drug regimen and had negative serology for syphilis and HIV.

Scientific literature fails to explain why most *T*. *gondii* seropositive patients have no ocular toxoplasmosis, describes very little about individuals with asymptomatic retinochoroidal lesion and is scarce in recurrence studies and susceptible individuals. The analysis of this population suggests an association between heterozygosis of IFN-γ+874T/A genotype, age and primary lesion and recurrence of TRC during the follow-up period in the studied population, after an active episode.

Further studies concerning host characteristics such as: cytokines profile, retinal micro-ambient age changes and its tolerance to *T*. *gondii* latency and persistency, immunological human senescence, as well as its interaction with parasite lineages can be the future challenge to understand recurrence and also occurrence of toxoplasmic retinochoroiditis.

## Conclusion

The analysis of this cohort showed that the risk of TRC recurrence during the first years after an active episode increased with age and was significantly greater in patients with primary lesion. This fact suggests that the elderly or individuals with primary lesions could benefit from prophylactic antimicrobials. In addition, this study suggests that the genetic polymorphism of IFN-γ +874T/A can be associated with TRC recurrence.
